# Comprehensive Clinical Characterization of Decade-Long Survivors of Metastatic Breast Cancer

**DOI:** 10.3390/cancers15194720

**Published:** 2023-09-25

**Authors:** Junghoon Shin, Ji-Yeon Kim, Jung Min Oh, Jeong Eon Lee, Seok Won Kim, Seok Jin Nam, Won Park, Yeon Hee Park, Jin Seok Ahn, Young-Hyuck Im

**Affiliations:** 1 Division of Hematology-Oncology, Department of Medicine, Samsung Medical Center, Seoul 06351, Republic of Korea; junghoon.shin@samsung.com (J.S.);; 2 Biomedical Research Institute, Samsung Medical Center, Seoul 06351, Republic of Korea; 3 School of Medicine, Sungkyunkwan University, Suwon 16419, Republic of Korea; 4 Division of Breast Surgery, Department of Surgery, Samsung Medical Center, Seoul 06351, Republic of Korea; 5 Department of Radiation Oncology, Samsung Medical Center, Seoul 06351, Republic of Korea

**Keywords:** chemotherapy, endocrine therapy, HER2, hormone receptor, metastatic breast cancer, survival, triple-negative breast cancer

## Abstract

**Simple Summary:**

Characterizing features of metastatic breast cancer (MBC) patients with exceptionally favorable prognoses may inform strategies to improve the survival of more typical patients. In this retrospective study, we analyzed clinicopathologic characteristics and treatments of 110 consecutive MBC patients who survived for >10 years. Approximately 70% of patients initially had a single-organ metastasis. The median duration of systemic therapy was 11, 8.4, 7.3, and 0.8 years in the hormone receptor-positive (HR+)/human epidermal growth factor receptor 2-negative (HER2−), HR+/HER2+, HR−/HER2+, and triple-negative breast cancer (TNBC) patients, respectively. Seven HER2+ and ten TNBC patients received <2 years of systemic therapy and remained alive and free of systemic therapy for >10 years. The TNBC subtype and local treatment with curative intent within 1 year of MBC diagnosis were associated with long-term treatment-free survival. Our study suggests that a small subset of patients with HER2+ MBC and metastatic TNBC may be curable with multimodality therapy.

**Abstract:**

Background: Elucidating the clinical features of metastatic breast cancer (MBC) patients with an exceptionally favorable prognosis may offer insights to improve the survival of more typical patients. Methods: We collected comprehensive real-world data on clinicopathologic characteristics, treatments, and outcomes of 110 consecutive MBC patients who survived for over ten years from the clinical data warehouse of Samsung Medical Center. Results: The cohort included 54 hormone receptor (HR)-positive/HER2-negative (HR+/HER2−), 21 HR+/HER2+, 16 HR−/HER2+, and 14 triple-negative breast cancer (TNBC) patients. The median age at MBC diagnosis was 48.5 years. Approximately 70% of patients initially had a single-organ metastasis. The most common site of metastasis was the lung (46.4%), followed by distant lymph nodes (37.3%). During a median follow-up of 14.6 years, the median duration of systemic therapy was 11, 8.4, 7.3, and 0.8 years in the HR+/HER2−, HR+/HER2+, HR−/HER2+, and TNBC subgroups, respectively. Seven HER2+ and ten TNBC patients received systemic treatment for less than two years and remained treatment-free for most of the follow-up period, suggesting a potential chance of cure. The TNBC subtype (*p* < 0.001) and local treatment with curative intent within 1 year of MBC diagnosis (*p* = 0.002) were significantly associated with long-term treatment-free survival. The survival of HER2+ MBC and TNBC patients, but not that of HR+/HER2− patients, plateaued approximately 13 years after MBC diagnosis. Conclusions: A small subset of patients with HER2+ MBC and metastatic TNBC may be curable with multimodality therapy. Prospective studies integrating clinical and genomic data may identify unique clinicogenomic features of MBC patients who can achieve durable disease control without prolonged chemotherapy.

## 1. Introduction

Metastatic solid tumors are generally considered incurable, with rare exceptions. Metastatic breast cancer (MBC) is not one of these exceptions. Although the progress made in treatments for MBC over the past three decades has translated into a significant population-level survival improvement, the 5-year relative survival of MBC remains around 30–40% [1,2]. Triple-negative breast cancer (TNBC) has the poorest prognosis among all breast cancer subtypes, with a median overall survival (OS) of approximately 1 year once the disease spreads to distant organs [3].

Nonetheless, real-world experience has consistently indicated that a small number of MBC patients have unusually deep responses to antineoplastic therapy and survive for over a decade [4,5,6,7]. Recent long-term follow-up data from the CLEOPATRA and MONALEESA-2 trials demonstrated that a subset of patients with human epidermal growth factor receptor 2 (HER2)-positive (HER2+) or hormone receptor (HR)-positive/HER2-negative (HR+/HER2−) MBC have durable responses that last for many years to the HER2−targeting agents plus chemotherapy or a cyclin-dependent kinase 4 and 6 (CDK4/6) inhibitor plus endocrine therapy (ET), respectively [8,9]. Growing evidence suggests that a small proportion of MBC patients may even be curable [10]. This evidence includes (1) the presence of pathologic complete response (CR) to neoadjuvant chemotherapy with or without targeted therapy [11], (2) the presence of survivors of MBC who achieve and maintain CR for over a decade after palliative chemotherapy or ET [5], and (3) the promising role of local therapy for eliminating the oligometastatic disease [12]. 

Understanding the clinical picture of prognostic outliers is crucial because it can provide a clue to clinical and biological features mediating therapeutic success. Previous studies on long-term survivors of MBC have yielded conflicting results on factors that predict this exceptional clinical outcome [5,13]. This discrepancy is likely due to the small number of cases included, the heterogeneity of study populations, and varying definitions of long-term survival. Long-term follow-up data of individual patients is essential for identifying and clinically characterizing long-term survivors. However, such data are scarce, as cases of long-term survivors with uninterrupted longitudinal data are rare. Most published data are case reports or small series of 10–20 patients, providing only limited insights on the determinants of such a favorable prognosis [13,14,15,16]. Here, we describe comprehensive, longitudinal, real-world data of clinicopathologic characteristics, treatment patterns, and outcomes of 110 MBC patients who survived for over a decade since their disease metastasized.

## 2. Materials and Methods

### 2.1. Eligible Patients and Data Collection

We identified all consecutive patients who (1) were diagnosed with pathologically confirmed breast carcinoma of stage IV according to the seventh edition of the American Joint Committee on Cancer staging manual between 1999 and 2012, (2) received at least one systemic treatment for metastatic disease, and (3) survived for longer than ten years since MBC diagnosis from the clinical data warehouse in Samsung Medical Center. Patients with stage I, II, or III breast cancer treated with curative intent and subsequently developed metastatic disease (i.e., recurrent MBC) were also included in the study. We obtained data regarding clinicopathologic characteristics, neoadjuvant and adjuvant treatments, breast cancer surgery, and local and systemic therapy for metastatic disease from the electronic medical records. The date of last survival follow-up or death was determined using the national health insurance database, which provides real-time information on the death status of virtually all domestic Korean citizens. To minimize information bias, the survival data of all patients still alive on 28 April 2023 were censored.

### 2.2. Pathologic Classification

Histologic diagnosis was determined based on pathology reports of pretreatment core-needle biopsies or surgical specimens of primary breast tumors. If these were unavailable, the diagnosis was made based on metastatic site biopsy. We defined estrogen receptor (ER) and progesterone receptor (PR) positivity as an Allred score of 3–8 assessed by standard immunohistochemistry (IHC) analysis on routine archival tissue samples [17]. Tumors that were positive for either ER or PR (or both) were considered HR-positive. HER2 positivity was defined as either HER2 IHC 3+ or *HER2* gene amplification detected by in situ hybridization methods according to the American Society of Clinical Oncology/College of American Pathologists HER2 testing guideline [18]. 

Since many patients had MBC that recurred after prior treatment for localized disease, we collected information on ER, PR, and HER2 status at initial diagnosis and at the time of initial metastasis. Molecular subtypes of patients with bilateral breast cancer (n = 4) were determined based on the tumor with a higher locoregional stage. We classified patients into four non-overlapping categories using HR and HER2 status information in the metastatic setting (if available) or initial diagnosis (only if metastatic setting information was not available): HR+/HER2−, HR+/HER2+, HR−/HER2+, and TNBC. Five patients had no information on HER2 status both at initial diagnosis and in the metastatic setting.

### 2.3. Statistical Analysis

We summarized the data as medians and ranges for continuous variables and numbers and percentages for discrete variables. The association between categorical variables was assessed using Fisher’s exact tests. The disease-free interval was defined as the time from the date of curative breast surgery to MBC diagnosis. The OS was defined as the time from MBC diagnosis to death from any cause. The duration of systemic treatment was defined as the total time spent receiving systemic therapy. The systemic treatment-free interval was defined as the sum of intervals between the end of each line of systemic treatment and the start of the following line. We estimated OS using the Kaplan–Meier method and follow-up time using the reverse Kaplan–Meier method. We used the log-log transformation of the survival function method to calculate 95% confidence intervals (CIs) for the Kaplan–Meier estimates [19]. We used the Cox proportional hazards regression analysis to estimate hazard ratios (HRs) and test for differences in OS between groups. All tests were two-tailed. We considered *p*-values less than 0.05 to be statistically significant. R version 4.2.2 was used for computation.

## 3. Results

### 3.1. Patient Characteristics

**Figure 1:**
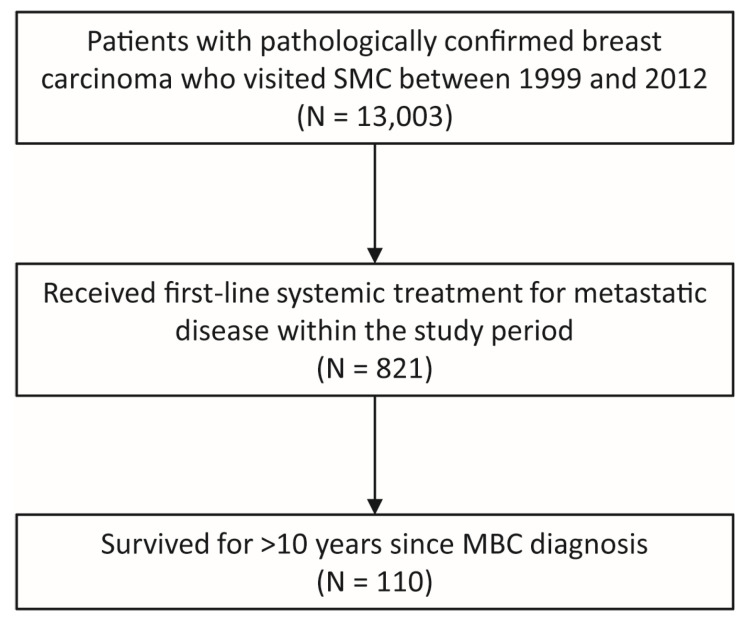
Patient selection.

We identified 110 patients who survived with MBC for over ten years, which we termed the long-term survivor (LTS) cohort (Figure 1). The LTS cohort included 105 patients that could be unambiguously classified into one of four breast cancer subtypes (i.e., HR+/HER2−, HR+/HER2+, HR−/HER2+, and TNBC). The remaining five patients had no information on HER2 status at initial diagnosis and in the metastatic setting. The clinicopathologic characteristics of patients are summarized in Table 1. The median age at diagnosis of MBC was 48.5 (range 26–69) in the LTS cohort and was slightly higher in the HR−/HER2+ subtype than the other three subtypes (Table 1). Most patients had metastatic disease before age 65. Approximately 20% of patients had de novo MBC in the HR+/HER2− and HER2+ subgroups, whereas all TNBC patients had recurrent MBC. Among the 90 patients with recurrent MBC, all but two (n = 88) underwent curative breast surgery. Seventy-five (85.2%) of these patients received neoadjuvant and/or adjuvant chemotherapy. Among the 35 patients whose breast cancer was initially HR+/HER2− and received curative breast surgery, 29 (82.9%) received adjuvant ET. The adjuvant ET regimens were tamoxifen (n = 23), toremifene (n = 3), anastrozole (n = 1), sequential use of tamoxifen and anastrozole (n = 1), and unknown (n = 1). The median disease-free interval was four years, ranging from 2.2 years in the HR−/HER2+ subgroup to 5.2 years in the HR+/HER2− subgroup. 

**Figure 2:**
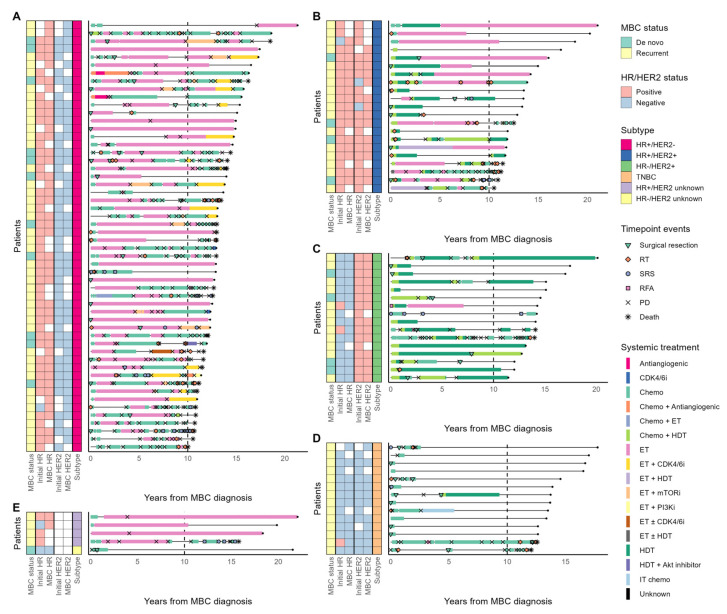
The clinical course of individual patients in the HR+/HER2− (**A**), HR+/HER2+ (**B**), HR−/HER2+ (**C**), TNBC (**D**), and HER2-unknown (**E**) subgroups. Empty boxes in the HR and HER2 status columns indicate missing information. The horizontal solid lines indicate the period of survival follow-up for each patient. Patients without the death symbol on the right end of the line were alive at the time of data cutoff. MBC, metastatic breast cancer; HR, hormone receptor; HER2, human epidermal growth factor receptor 2; TNBC, triple-negative breast cancer; RT, radiation therapy; SRS, stereotactic radiosurgery; RFA, radiofrequency ablation; PD, progressive disease; CDK4/6i, cyclin-dependent kinase 4 and 6 inhibitor; chemo, chemotherapy; ET, endocrine therapy; HDT, HER2-directed therapy; mTORi, mammalian target of rapamycin inhibitor; PI3Ki, phosphoinositide 3-kinase inhibitor; IT chemo, intrathecal chemotherapy.

There was no significant difference in HR and HER2 status between the time of initial diagnosis and the time of initial metastasis. Five patients experienced a phenotypic change in their HR and HER2 status, with two switching from HR+/HER2− to HR+/HER2+, one from HR+/HER2− to TNBC, and two from HR+/HER2+ to HR−/HER2+. Consistent with the general breast cancer population in Korea, 22 (59.5%) patients with HER2+ MBC were HR-positive at initial diagnosis, and 16 (43.2%) were still HR-positive when the disease metastasized (Figure 2) [2].

Approximately 70% of patients initially had metastasis in a single organ, with no significant variation between subtypes (Table 1). The lung was the most common site of metastasis in both HR+/HER2− and TNBC subgroups, affecting approximately 60% of patients, while the distant lymph node-only relapse was more common in the HER2+ subgroups. The most commonly involved distant nodal groups included mediastinal, cervical, and hilar lymph nodes (Supplementary Table S1). Visceral organ metastasis was present in about two-thirds of patients with HR+/HER2− MBC or TNBC but in less than half of patients with HER2+ MBC (Table 1). 

### 3.2. Systemic Treatment for MBC

**Figure 3:**
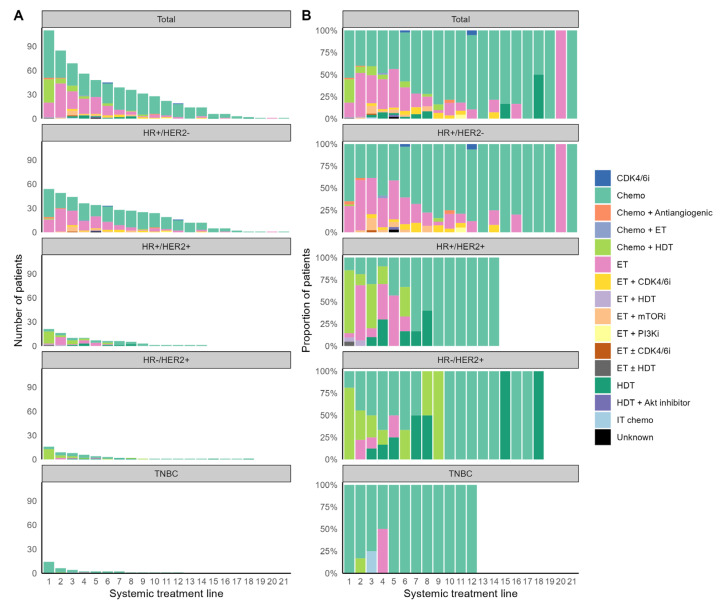
The numbers (**A**) and proportions (**B**) of patients who received systemic therapy of lines 1 to 21. Abbreviations are as in Figure 2. For the specific regimens used, see Supplementary Table S3. For the regimen classification rule, see Supplementary Table S4.

Patients in the LTS cohort received a median of four lines of systemic treatment. All patients received at least one systemic therapy, with a gradually decreasing number of patients who received subsequent therapies (Figure 3A). Patients with HR+/HER2− and HER2+ MBC received longer durations of systemic therapy than patients with TNBC, likely due to the better tolerability of ET and HER2-targeting agents than cytotoxic chemotherapy and different tumor biology (Figure 2). The median duration of systemic treatment was 11 (range, 2.5–17.2), 8.4 (range, 0.5–20.6), 7.3 (range, 0.9–16.8), and 0.8 (range, 0.3–11) years for the HR+/HER2−, HR+/HER2+, HR−/HER2+, and TNBC subgroups, respectively. 

Cytotoxic chemotherapy alone was the most common first-line treatment for both HR+/HER2− and TNBC subgroups, with 35 (64.8%) and 14 (100%) patients receiving it as initial treatment, respectively, whereas 15 (71.4%) patients with HR+/HER2+ MBC and 13 (81.2%) patients with HR−/HER2+ MBC received a frontline combination of chemotherapy and HER2-directed therapy (Figure 3B). All except one patient with HR+/HER2− MBC received at least one ET for metastatic disease, and 16 (29.6%) of them received it in the first line. An HR+/HER2− patient who did not receive any ET had left cerebellum and lung metastases at the initial recurrence of breast cancer, underwent surgical tumor removal followed by stereotactic radiosurgery (SRS) for the brain metastasis, and then received sequential lines of chemotherapy with docetaxel (for 3 months) and capecitabine (for 44 months). After two additional SRS and whole-brain radiation therapy to control the intracranial disease progression, the patient remained treatment-free and alive for approximately nine years. While systemic treatments used for the HR+/HER2− MBC were primarily ET alone or combined with targeted agents, 17 HR+/HER2− patients received more chemotherapy than ET in terms of treatment duration (Supplementary Figure S1). We found that patients with HR+ MBC with HER2 IHC score of 0 (HR+/HER2−zero) received more ET than chemotherapy compared to patients with HR+ MBC with HER2 IHC scores of 1 or 2 (HR+/HER2−low; *p* = 0.011; Supplementary Table S2). Fourteen (66.6%) patients with HR+/HER2+ MBC also received ET, mostly in the second or later line. Among them, ten patients received ET for longer than HER2-directed therapy with or without chemotherapy (Figure 2).

Doxorubicin plus cyclophosphamide was the most common first-line treatment for both HR+/HER2− and TNBC subtypes, with 20 (37%) and 4 (28.6%) patients receiving it, respectively, whereas 15 (71.4%) patients with HR+/HER2+ MBC and 12 (75%) with HR−/HER2+ MBC received taxane plus trastuzumab for the first-line treatment. Notably, five (31.3%) patients with HR−/HER2+ MBC discontinued HER2-directed therapy within five years of MBC diagnosis and remained progression-free for over ten years until the end of follow-up (Figure 2). All systemic treatment regimens used from the first to the third line for each molecular subtype are summarized in Supplementary Table S3. The regimen classification rule is summarized in Supplementary Table S4.

### 3.3. Local Treatment for Metastatic Disease

Seventy-five (68.2%) patients received local treatment after diagnosis of MBC, including surgical resection (n = 55 (50%)), radiation therapy (n = 42 (38.2%)), SRS (n = 15 (13.6%)), and radiofrequency ablation (n = 1 (0.9%)). The proportion of patients who received local treatment was slightly higher in HER2+ and TNBC subtypes than in the HR+/HER2− subtype (Table 2). Of note, 37 (33.6%) patients received one or more local treatments with curative intent (i.e., local treatments excluding palliative radiation therapy) within 1 year of MBC diagnosis, including surgical tumor removal (n = 35), SRS for intracranial metastasis (n = 4), and radiofrequency ablation for liver metastasis (n = 1). The most common anatomical sites of tumors resected within a year of MBC diagnosis was the lung (n = 12), followed by the breast (n = 10; Supplementary Table S5). Breast tumor excision in the setting of metastatic disease was performed in five (three HR+/HER2−, one HR+/HER2+, and one HR−/HER2+) patients before any systemic treatment for MBC (i.e., upfront primary tumor resection) in four (one HR+/HER2−, one HR+/HER2+, one HR−/HER2+, and one TNBC) patients who had metastatic disease controlled with the first-line systemic therapy, and in one patient with HR−/HER2-unknown MBC who had a primary tumor that was refractory to two lines of systemic therapy.

Patients with TNBC were more likely to receive local treatment with curative intent within 1 year of MBC diagnosis than patients with HR+/HER2− or HER2+ disease (*p* = 0.016). Nine (64.3%) of the 14 patients with TNBC underwent surgical tumor removal within 1 year of MBC diagnosis. They received short periods of perioperative systemic therapy and were free of systemic treatment for a median of 13.1 (range, 8.5–16.7) years thereafter. Specifically, six patients had lung metastases resected, and one each had tumors resected from the brain, chest wall, and breast. Two TNBC patients also received SRS for metastatic brain tumors within a month of MBC diagnosis and never had intracranial disease progression after that. 

### 3.4. Patients with a Long Systemic Treatment-Free Interval

**Figure 4:**
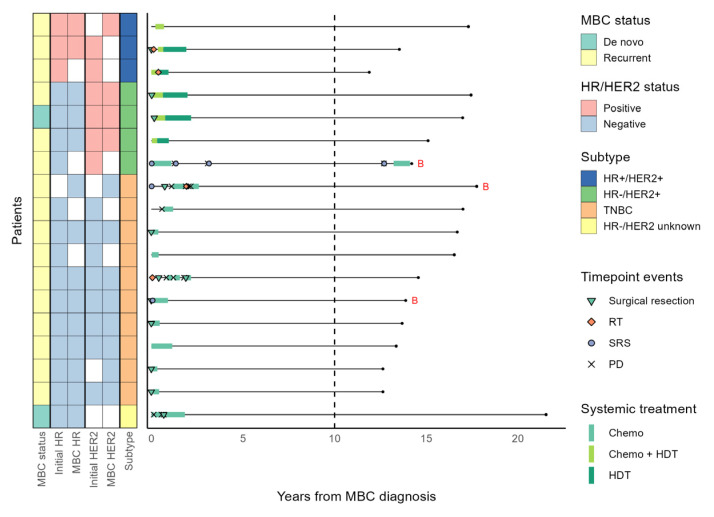
The clinical course of patients who received systemic treatment for less than two years throughout the follow-up period. Patients denoted with a red ‘B’ symbol at the right end of the horizontal line had brain metastasis at diagnosis of MBC. Abbreviations are as in Figure 2.

The median systemic treatment-free interval was 2.5, 3, 6.9, and 12.7 years in the HR+/HER2−, HR+/HER2+, HR−/HER2+, and TNBC subtypes, respectively. Eighteen patients in the LTS cohort received systemic treatment for less than two years and remained treatment-free for most of the follow-up period, including three HR+/HER2+, four HR−/HER2+, and ten TNBC patients (Figure 4). After initial short courses of chemotherapy with or without HER2-directed therapy, all these patients were off systemic treatment for over ten years. Among these patients, only one with HR−/HER2+ MBC experienced cancer progression (localized to the brain) after cessation of systemic treatment, which was successfully controlled with sequential SRS (Figure 4). A prespecified association analysis revealed that the molecular subtype of breast cancer (*p* < 0.001) and local treatment with curative intent within 1 year of MBC diagnosis (*p* = 0.002) were significantly associated with a systemic treatment duration of less than two years (Table 3). 

Three of four patients with brain metastases at the time of MBC diagnosis received less than two years of systemic treatment. At the time of initial metastasis, an HR−/HER2+ MBC patient had three metastatic brain lesions, and two TNBC patients had one brain metastasis. All three patients received upfront SRS before any systemic treatment for MBC, and one TNBC patient received subsequent surgical removal of the remnant metastatic brain lesion. The other TNBC patient also had a single 10 mm lung metastasis at MBC diagnosis and received upfront pulmonary wedge resection (Figure 4).

### 3.5. Survival

**Figure 5:**
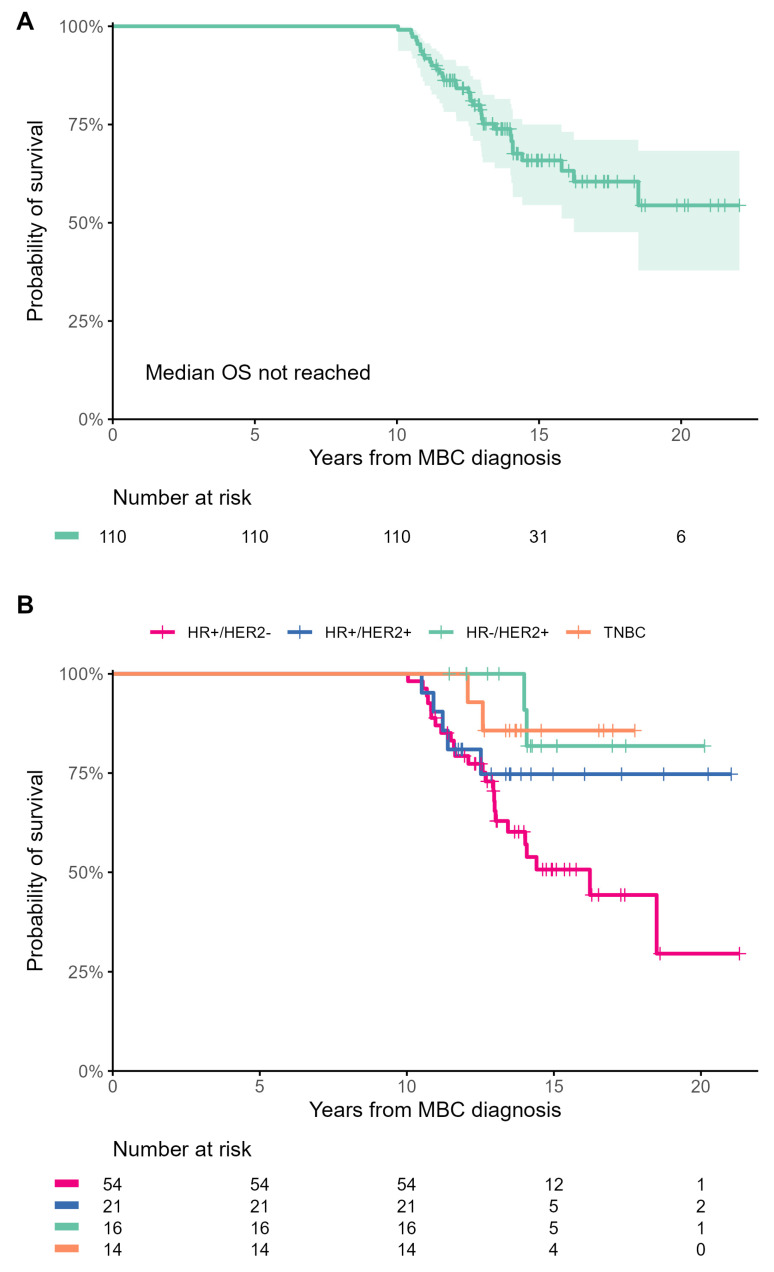
Kaplan–Meier estimates for overall survival in the entire cohort (**A**) and patients of each breast cancer subtype (**B**).

After a median follow-up of 14.6 years, 34 (30.9%) patients died. The median OS was not reached in the LTS (Figure 5A), HR+/HER2+, HR−/HER2+, and TNBC cohorts, while it was 16.2 years in the HR+/HER2− cohort (Figure 5B). After the 10-year landmark, patients with HER2+ MBC had significantly longer OS than patients with HR+/HER2− MBC (HR, 0.37; 95% CI, 0.16–0.86, *p* = 0.021). Visual inspection of survival curves also showed a trend toward longer OS of the TNBC subgroup compared to the HR+/HER2− subgroup, but this did not reach statistical significance due to the small number of patients with TNBC (HR, 0.27; 95% CI, 0.06–1.14, *p* = 0.075). After around 13 years from MBC diagnosis, the Kaplan–Meier survival curves for HR+/HER2+, HR−/HER2+, and TNBC subgroups reached a plateau, indicating the potential for cure in these subtypes. However, this was not observed in the HR+/HER2− subgroup (Figure 5B).

In the LTS cohort, four patients had brain metastases at the time of breast cancer recurrence, including three patients described above. One patient with HR+/HER2− MBC had a single metastatic tumor in the left cerebellum and a single 16 mm lung metastasis at MBC diagnosis. This patient received upfront surgical resection of the brain tumor followed by SRS for the remnant lesion one month later, along with systemic chemotherapy with docetaxel. All four patients were alive at the time of data cutoff, with the censored OS ranging from 12.9 to 17.8 years. One patient with TNBC developed leptomeningeal metastasis (proven by cytologic evaluation) approximately 31 months after MBC diagnosis. This patient received 95 intrathecal injections of methotrexate over three years and remained alive 11 years after the first detection of leptomeningeal disease.

## 4. Discussion

This study reports the largest published cohort of MBC patients with over ten years of survival. The comprehensive clinicopathologic profiles, systemic and local treatment history, and outcomes of the LTS cohort characterized in this study can help identify patients with MBC who are most likely to have an exceptionally favorable prognosis and inform the optimal sequence of therapies that should be personalized for each patient. The exceptional nature of the LTS cohort is further accentuated by the fact that most patients in this cohort, especially during the early period of treatment for MBC, had no access to novel therapies such as CDK4/6 inhibitors, pertuzumab, trastuzumab emtansine, and trastuzumab deruxtecan that have significantly improved the prognosis of MBC in recent years [8,9,20,21]. We found that (1) a small number of HER2+ MBC and TNBC patients were able to discontinue systemic treatment and remain treatment-free and alive for over ten years, and (2) long-term treatment-free survival is primarily determined by the ability to control the primary and oligometastatic lesions with local therapies in combination with a relatively short duration of systemic treatment. Our findings suggest that a small subset of patients with HER2+ MBC and TNBC may be cured with a multimodality approach. Therefore, it may be clinically useful to divide MBC patients into prognostically distinct subgroups based on the feasibility of local treatment to eliminate oligometastatic disease and consideration of their tumor biology, as is already standard in other solid cancers [22,23]. 

The LTS cohort was younger and had a distinct molecular composition than the general breast cancer population of MBC [1,2,24,25]. The most common molecular subtype in the LTS cohort, HR+/HER2−, accounted for just half of the patients, compared to the 60–70% proportion consistently seen in more general MBC cohorts [24,25]. This gap was filled by the relative overrepresentation of HER2+ tumors in the LTS cohort. Since trastuzumab revolutionized the treatment landscape and survival for HER2+ MBC in the early 2000s [26], this biased subtype composition is unsurprising. On the other hand, nearly 80% of HR+/HER2− patients had been exposed to adjuvant tamoxifen. In this subtype, the median disease-free interval of 5.2 years indicates that over half of the patients had acquired primary or secondary endocrine resistance during the adjuvant phase. Most HR+/HER2− patients began systemic treatment for MBC before the era of CDK4/6 inhibitors. Accordingly, approximately 70% of HR+/HER2− patients received frontline chemotherapy instead of ET (Supplementary Table S3). The relatively higher frequency of visceral disease at presentation may also explain the shorter survival of HR+/HER- patients than HER2+ patients in the LTS cohort. 

Tumor growth is accompanied by clonal evolution, which can produce subclones resistant to therapy [27]. Breast cancer is known for its high degree of genomic heterogeneity [28], with TNBC having the highest mutation rate [29]. The highly mutagenic nature of TNBC may explain why the prognosis of this devastating disease has not improved as much as that of HR+ or HER2+ subtypes over the past three decades [30]. Therefore, earlier treatment, when the tumor has undergone fewer cell divisions and acquired fewer mutations, may provide a better chance of cure. Our data support this hypothesis. Most TNBC patients in the LTS cohort had an oligometastatic disease at the time of diagnosis, and approximately two-thirds of them had surgical excision of metastatic tumors early in the treatment course. As of the data cutoff, all these patients were off treatment and alive for over eight years. Earlier trials found no survival benefit for intensive surveillance in patients with curatively resected localized breast cancer [31,32]. However, our data suggest a well-designed, individualized approach according to risk stratification for surveillance may be worth revisiting, particularly for HER2+ and TNBC subtypes.

Approximately 30% of HR+/HER2− MBC patients in the LTS cohort received cytotoxic chemotherapy for longer than ET, and these patients were enriched with HER2-low MBC. Mounting evidence suggests that HER2-low and HER2-zero breast cancers might be different disease entities [33,34]. Recently, the DESTINY-Breast04 trial showed that trastuzumab deruxtecan significantly improved PFS and OS in HR+/HER2−low MBC patients compared to the physician’s choice of chemotherapy [35]. While the predictive role of HER2-low status on ET response is not firmly established, our data suggest that HR+/HER2−low MBC may derive less benefit from ET than HR+/HER2−zero MBC. Recent retrospective studies also reported consistent results suggesting bidirectional crosstalk between the HR and HER2 pathways as a potential mechanism of hormonal resistance and poor outcomes [36,37].

Our longitudinal follow-up data revealed significant heterogeneity in treatment patterns for HR+/HER2+ MBC: approximately half of the patients received ET for longer than HER2-directed therapy, while others received HER2-directed therapy for longer than ET. In addition, a small number of patients with HR−/HER2+ MBC discontinued HER2-directed maintenance therapy and never had disease progression. Accurate identification of patients who can achieve long-term disease control with chemotherapy-free treatment (i.e., ET with or without HER2-directed therapy) and those who can safely discontinue HER2-directed maintenance therapy is essential to reduce treatment-related toxicity and improve the outcome of HER2+ MBC. The small number of HER2+ cases in the LTS cohort warrants future studies specifically designed to identify patient- and treatment-related factors associated with different clinical courses of HER2+ MBC.

The data presented in this study are rich but should be interpreted with caution due to the study’s retrospective nature. Most patients were diagnosed and began treatment before the advent of novel targeted therapies such as CDK4/6 inhibitors, pertuzumab, trastuzumab emtansine, and trastuzumab deruxtecan. The increasing availability of these highly efficacious and less toxic agents may diminish the role of local treatment modalities for metastatic disease. The absence of a contemporaneous control cohort at the opposite extreme of the survival distribution also limited statistical assessment of potential prognostic factors. Although the national health insurance database provided complete survival data for nearly all patients in the study, data on treatments and responses were incomplete for over two years in 11% of patients, primarily due to patients transferring to other institutions. In addition, we did not systematically assess comorbidities, such as congestive heart failure or interstitial lung disease, that could have affected the choice of breast cancer therapy and survival. Lastly, we cannot exclude the possibility of selection bias because all patients were identified from a single institution database.

Nonetheless, the LTS cohort is the largest group of decade-long survivors of MBC ever published and provides an invaluable resource for understanding patient and treatment factors that drive unusual therapeutic success in MBC. The molecular mechanisms that mediate the exceptional therapeutic response are now beginning to be elucidated [38]. The Exceptional Responders Initiative set a milestone in these efforts and proposed plausible mechanisms in ~23% of patients included in the study [39]. On the other hand, the inability to infer a mechanistic explanation for ~77% of patients, even with analysis of multi-platform genomic data, leaves ample room for future studies that integrate clinical and genomic data to delineate the interaction between these different layers of information. Such studies may reveal unique clinicogenomic features of long-term survivors that can be exploited therapeutically and ultimately be used to improve outcomes of the more general breast cancer population.

## 5. Conclusions

Patients with MBC may have exceptionally favorable prognoses regardless of the molecular subtype. A small subset of patients with oligometastatic MBC may be curable with multimodality therapy. Future studies that integrate clinical and genomic data may identify unique clinicogenomic characteristics of MBC patients who can achieve durable disease control after receiving only a short course of systemic therapy.

## Figures and Tables

**Table 1:** Patient characteristics.

Characteristic	Total (n = 110)	HR+/HER2− (n = 54)	HR+/HER2+ (n = 21)	HR−/HER2+ (n = 16)	TNBC (n = 14)
Year of MBC diagnosis, n (%)					
1999–2002	10 (9.1%)	6 (11.1%)	1 (4.8%)	0 (0%)	0 (0%)
2003–2006	33 (30%)	15 (27.8%)	6 (28.6%)	5 (31.3%)	5 (35.7%)
2007–2010	53 (48.2%)	26 (48.1%)	10 (47.6%)	8 (50%)	9 (64.3%)
2011–2012	14 (12.7%)	7 (13%)	4 (19%)	3 (18.8%)	0 (0%)
Age at diagnosis of MBC					
Median (range)	48.5 (26–69)	47 (35–69)	48 (26–68)	54.5 (31–63)	48.5 (29–67)
<50, n (%)	59 (53.6%)	30 (55.6%)	14 (66.7%)	6 (37.5%)	7 (50%)
50–64, n (%)	41 (37.3%)	20 (37%)	4 (19%)	10 (62.5%)	4 (28.6%)
≥65, n (%)	10 (9.1%)	4 (7.4%)	3 (14.3%)	0 (0%)	3 (21.4%)
Disease status at MBC diagnosis, n (%)					
De novo	20 (18.2%)	11 (20.4%)	4 (19.0%)	4 (25.0%)	0 (0%)
Recurrent ^a^	90 (81.8%)	43 (79.6%)	17 (81.0%)	12 (75.0%)	14 (100%)
Stage at initial diagnosis of BC, n (%) ^b^					
I	20 (18.2%)	7 (13.0%)	7 (33.3%)	2 (12.5%)	2 (14.3%)
II	34 (30.9%)	20 (37.0%)	6 (28.6%)	4 (25.0%)	3 (21.4%)
III	21 (19.1%)	10 (18.5%)	2 (9.5%)	5 (31.3%)	4 (28.6%)
IV	20 (18.2%)	11 (20.4%)	4 (19.0%)	4 (25.0%)	0 (0%)
Unknown	15 (13.6%)	6 (11.1%)	2 (9.5%)	1 (6.3%)	5 (35.7%)
Prior neoadjuvant or adjuvant treatment, n (%) ^c^					
Both neoadjuvant and adjuvant chemotherapy	9 (10.2%)	3 (7.1%)	2 (12%)	1 (8%)	3 (23.1%)
Adjuvant chemotherapy only	66 (75%)	34 (81%)	9 (53%)	10 (83%)	9 (69.2%)
Adjuvant endocrine therapy	55 (62.5%)	32 (76.2%)	13 (76%)	5 (42%)	3 (23.1%)
Adjuvant radiation therapy	52 (59.1%)	20 (47.6%)	11 (65%)	10 (83%)	10 (76.9%)
Disease-free interval (years)					
Median (range)	4 (0.2–28.9)	5.2 (0.5–28.9)	2.5 (0.2–8.6)	2.2 (0.9–7.9)	3.9 (1.1–8.5)
<1 year, n (%)	7 (6.4%)	2 (3.7%)	3 (14.3%)	2 (12.5%)	0 (0%)
1–2 years, n (%)	11 (10.0%)	1 (1.9%)	4 (19.0%)	3 (18.8%)	3 (21.4%)
≥2 years, n (%)	70 (63.6%)	39 (72.2%)	10 (47.6%)	7 (43.8%)	10 (71.4%)
Did not receive curative breast surgery, n (%)	22 (20.0%)	12 (22.2%)	4 (19.0%)	4 (25.0%)	1 (7.1%)
Histologic diagnosis, n (%)					
Invasive ductal carcinoma	96 (87.3%)	46 (85.2%)	19 (90.5%)	14 (87.5%)	13 (92.9%)
Invasive lobular carcinoma	1 (0.9%)	1 (1.9%)	0 (0%)	0 (0%)	0 (0%)
Invasive micropapillary carcinoma	3 (2.7%)	0 (0%)	1 (4.8%)	1 (6.3%)	1 (7.1%)
Invasive cribriform carcinoma	2 (1.8%)	1 (1.9%)	1 (4.8%)	0 (0%)	0 (0%)
Invasive apocrine carcinoma	1 (0.9%)	0 (0%)	0 (0%)	0 (0%)	0 (0%)
Invasive tubulolobular carcinoma	1 (0.9%)	1 (1.9%)	0 (0%)	0 (0%)	0 (0%)
Mucinous carcinoma	2 (1.8%)	2 (3.7%)	0 (0%)	0 (0%)	0 (0%)
Signet ring cell carcinoma	1 (0.9%)	0 (0%)	0 (0%)	1 (6.3%)	0 (0%)
Adenocarcinoma ^d^	2 (1.8%)	2 (3.7%)	0 (0%)	0 (0%)	0 (0%)
Unknown	1 (0.9%)	1 (1.9%)	0 (0%)	0 (0%)	0 (0%)
ER Allred score ^e^					
0–2	35 (31.8%)	1 (1.9%) ^f^	2 (9.5%) ^g^	16 (100%)	14 (100%)
3–5	15 (13.6%)	7 (13.0%)	7 (33.3%)	0 (0%)	0 (0%)
6–8	58 (52.7%)	45 (83.3%)	12 (57.1%)	0 (0%)	0 (0%)
Unknown	2 (1.8%)	1 (1.9%)	0 (0%)	0 (0%)	0 (0%)
PR Allred score ^e^					
0–2	54 (49.1%)	12 (22.2%)	8 (38.1%)	16 (100%)	14 (100%)
3–5	15 (13.6%)	9 (16.7%)	6 (28.6%)	0 (0%)	0 (0%)
6–8	39 (35.5%)	32 (59.3%)	7 (33.3%)	0 (0%)	0 (0%)
Unknown	2 (1.8%)	1 (1.9%)	0 (0%)	0 (0%)	0 (0%)
HER2 IHC score ^e^					
0	45 (40.9%)	33 (61.1%)	0 (0%)	0 (0%)	12 (85.7%)
1	15 (13.6%)	13 (24.1%)	1 (4.8%) ^h^	0 (0%)	1 (7.1%)
2	18 (16.4%)	4 (7.4%)	6 (28.6%)	5 (31.3%)	1 (7.1%)
3	25 (22.7%)	0 (0%)	14 (66.7%)	11 (68.8%)	0 (0%)
Unknown	7 (6.4%)	4 (7.4%)	0 (0%)	0 (0%)	0 (0%)
Number of metastatic organs at MBC diagnosis, n (%)					
1	78 (70.9%)	35 (64.8%)	17 (81.0%)	11 (68.8%)	10 (71.4%)
≥2	32 (29.1%)	19 (35.2%)	4 (19.0%)	5 (31.3%)	4 (28.6%)
Metastatic sites at MBC diagnosis, n (%)					
Lung	51 (46.4%)	33 (61.1%)	6 (28.6%)	3 (18.8%)	8 (57.1%)
Distant lymph node	41 (37.3%)	16 (29.6%)	10 (47.6%)	9 (56.3%)	5 (35.7%)
Bone	33 (30.0%)	18 (33.3%)	6 (28.6%)	4 (25.0%)	2 (14.3%)
Liver	15 (13.6%)	10 (18.5%)	1 (4.8%)	3 (18.8%)	1 (7.1%)
Brain	4 (3.6%)	1 (1.9%)	0 (0%)	1 (6.3%)	2 (14.3%)
Others	13 (11.8%)	8 (14.8%)	2 (9.5%)	2 (12.5%)	1 (7.1%)
Visceral ^i^	66 (60.0%)	39 (72.2%)	8 (38.1%)	8 (50.0%)	10 (71.4%)

^a^ Patients with initially nonmetastatic breast cancer who did not undergo curative surgery and subsequently developed metastatic disease were also counted as having recurrent MBC. ^b^ Patients who received preoperative systemic treatment were classified based on the initial clinical stage. ^c^ Proportions are based on the number of patients who received curative breast surgery (n = 88) as a denominator. ^d^ Only information on metastatic site biopsy was available. ^e^ IHC scores were determined based on tissues obtained after the diagnosis of MBC if available, and tissues obtained at initial diagnosis otherwise. ^f^ This patient was ER-negative but PR-positive at the diagnosis of MBC. ^g^ One of these two patients was ER-negative but PR-positive at the diagnosis of MBC. The other patient was ER-negative at initial breast cancer diagnosis and ER-positive at MBC diagnosis. However, the ER Allred score information was only available for the initial diagnosis specimen and not for the metastatic setting specimen. Therefore, this patient was categorized into the ER Allred score range of 0–2, although she had HR+/HER2+ MBC. ^h^ This patient’s initial diagnosis specimen had an HER2 IHC score of 1+. No IHC retesting was performed at MBC recurrence, but the *HER2* fluorescence in situ hybridization test indicated a HER2/CEP17 copy number ratio of 4.22 and an average *HER2* copy number of 7.09, which is diagnostic of HER2-positive disease. Hence, this patient was classified as HER2+ MBC. ^i^ Visceral metastases were defined by lesions involving the following locations: lung, liver, bowel, pancreas, adrenal gland, pleura, pericardium, peritoneum, and CNS. HR, hormone receptor; HER2, human epidermal growth factor receptor 2; TNBC, triple-negative breast cancer; MBC, metastatic breast cancer; BC, breast cancer; ER, estrogen receptor; PR, progesterone receptor; IHC, immunohistochemistry; CEP17, centromere enumeration probe 17.

**Table 2:** Patients who received local treatments.

Local Treatment, n (%)	Anytime				Within a Year of MBC Diagnosis			
	HR+/HER2−	HR+/HER2+	HR−/HER2+	TNBC	HR+/HER2−	HR+/HER2+	HR−/HER2+	TNBC
Any	33 (61.1%)	17 (81%)	12 (75%)	11 (78.6%)	18 (33.3%)	11 (52.4%)	6 (37.5%)	10 (71.4%)
Surgical resection	25 (46.3%)	10 (47.6%)	9 (56.2%)	10 (71.4%)	15 (27.8%)	7 (33.3%)	3 (18.8%)	9 (64.3%)
Radiation therapy	20 (37%)	12 (57.1%)	4 (25%)	5 (35.7%)	6 (11.1%)	7 (33.3%)	2 (12.5%)	3 (21.4%)
Stereotactic radiosurgery	8 (14.8%)	2 (9.5%)	2 (12.5%)	2 (14.3%)	1 (1.9%)	0 (0%)	1 (6.2%)	2 (14.3%)
Radiofrequency ablation	0 (0%)	0 (0%)	1 (6.2%)	0 (0%)	0 (0%)	0 (0%)	1 (6.2%)	0 (0%)

MBC, metastatic breast cancer; HR, hormone receptor; HER2, human epidermal growth factor receptor 2; TNBC, triple-negative breast cancer.

**Table 3:** Clinical factors associated with systemic treatment duration shorter than 2 years in the LTS cohort.

Variable	Systemic Treatment Duration < 2 Years (n = 18)	Systemic Treatment Duration ≥ 2 Years (n = 92)	*p*-Value
Year at MBC diagnosis, n (%)			0.71
1999–2002	1 (5.6%)	9 (9.8%)	
2003–2006	7 (38.9%)	26 (28.3%)	
2007–2010	9 (50.0%)	44 (47.8%)	
2011–2012	1 (5.6%)	13 (14.1%)	
Disease status at MBC diagnosis, n (%)			0.519
De novo	2 (11.1%)	18 (19.6%)	
Recurrent	16 (88.9%)	74 (80.4%)	
Disease-free interval (years), n (%)			0.054
<1 year	0 (0%)	7 (7.6%)	
1–2 years	5 (27.8%)	6 (6.5%)	
≥2 year	11 (61.1%)	59 (64.1%)	
Did not receive curative breast surgery	2 (11.1%)	20 (21.7%)	
Molecular subtype			<0.001
HR+/HER2−	0 (0%)	54 (58.7%)	
HR+/HER2+	3 (16.7%)	18 (19.6%)	
HR−/HER2+	4 (22.2%)	12 (13.0%)	
TNBC	10 (55.6%)	4 (4.3%)	
HR+/HER2 unknown	0 (0%)	4 (4.3%)	
HR−/HER2 unknown	1 (5.6%)	0 (0%)	
Number of metastatic organs at MBC diagnosis, n (%)			0.777
1	12 (66.7%)	66 (71.7%)	
≥2	6 (33.3%)	26 (28.3%)	
Curative local treatment within a year of MBC diagnosis			0.002
Done	12 (66.7%)	25 (27.2%)	
Not done	6 (33.3%)	67 (72.8%)	

LTS, long-term survivors; MBC, metastatic breast cancer; HR, hormone receptor; HER2, human epidermal growth factor receptor 2; TNBC, triple-negative breast cancer.

## Data Availability

All data generated or analyzed during this study are either included in this article or available from the first author or the corresponding author upon request.

## Supplementary Materials

The following supporting information can be downloaded at: https://www.mdpi.com/article/10.3390/cancers15194720/s1, Figure S1: Fraction of the total time spent on receiving each category of systemic treatment in patients with HR+/HER2− MBC; Table S1: Involved nodal areas of patients with distant lymph node metastasis at MBC diagnosis; Table S2: Baseline clinical factors associated with systemic treatment primarily with ET vs. chemotherapy in the HR+/HER2− cohort; Table S3: Systemic treatment regimens used from the first to the third line in the LTS cohort; Table S4: Systemic treatment regimen classification; Table S5: Anatomical sites of tumors excised within a year after diagnosis of MBC.
