# Supplementary material for: Comprehensive Clinical Characterization of Decade-Long Survivors of Metastatic Breast Cancer
Source: Cancers (Basel). 2023 Sep 25;15(19):4720. doi: 10.3390/cancers15194720 (PMC10571750; doi:10.3390/cancers15194720)
Supplement: Supplementary file 1 [file cancers-15-04720-s001.zip › cancers-2619070-supplementary-0926/Supplementary_Figure_S1_Cancers.pdf]

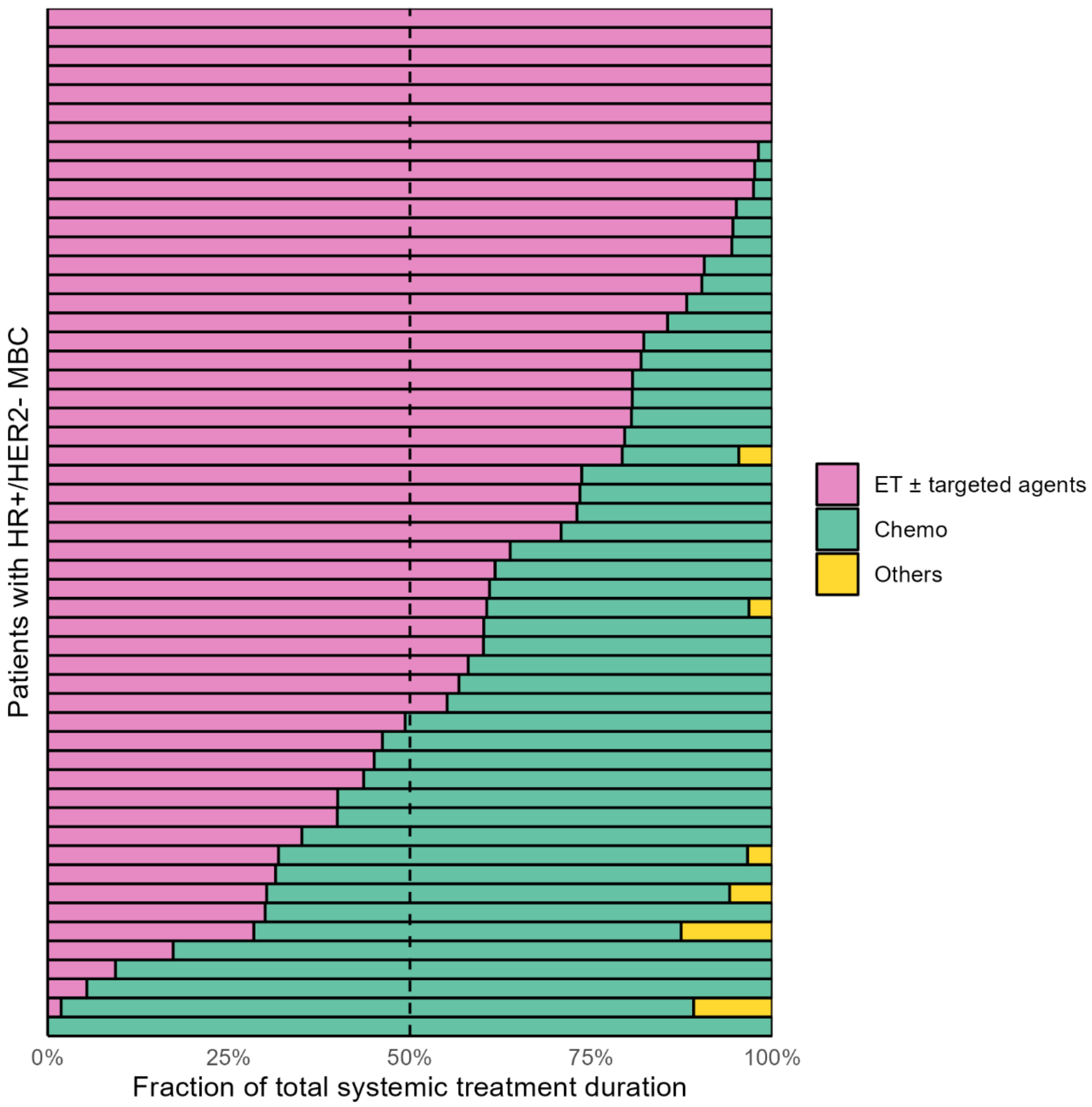


**Supplementary Figure S1.** Fraction of the total time spent on receiving each category of systemic treatment in patients with HR+/HER2- MBC. Abbreviations are as in Figure 2.
